# A Fusion Model Based on Dynamic Web Browsing Behavior Analysis for IoT Insider Threat Detection

**DOI:** 10.3390/s22176471

**Published:** 2022-08-28

**Authors:** Jiarong Wang, Junyi Liu, Tian Yan, Mingshan Xia, Jianshu Hong, Caiqiu Zhou

**Affiliations:** Institute of High Energy Physics, Chinese Academy of Sciences, 19B Yuquan Road, Shijingshan District, Beijing 100049, China

**Keywords:** Internet of things, insider threat, web browsing behavior, anomaly detection

## Abstract

With the wide application of Internet of things (IoT) devices in enterprises, the traditional boundary defense mechanisms are difficult to satisfy the demands of the insider threats detection. IoT insider threat detection can be more challenging, since internal employees are born with the ability to escape the deployed information security mechanism, such as firewalls and endpoint protection. In order to detect internal attacks more accurately, we can analyze users’ web browsing behaviors to identify abnormal users. The existing web browsing behavior anomaly detection methods ignore the dynamic change of the web browsing behavior of the target user and the behavior consistency of the target user in its peer group, which results in a complex modeling process, low system efficiency and low detection accuracy. Therefore, the paper respectively proposes the individual user behavior model and the peer-group behavior model to characterize the abnormal dynamic change of user browsing behavior and compare the mutual behavioral inconsistency among one peer-group. Furthermore, the fusion model is presented for insider threat detection which simultaneously considers individual behavioral abnormal dynamic changes and mutual behavioral dynamic inconsistency from peers. The experimental results show that the proposed fusion model can accurately detect insider threat based on the abnormal user web browsing behaviors in the enterprise networks.

## 1. Introduction

With the significant growth of Internet of things (IoT) devices, the IoT system has been further developed and extended [1]. The interaction forms between people and devices are more abundant than ever before [2,3]. More and more companies use various types of IoT devices, such as webcams, data collection sensors, network printers, etc., to make office more convenient. At the same time, by using data acquisition sensors, employees can monitor the operation of automatic assembly lines through network browsing without visiting the factory.

The heterogeneity and diversity of IoT devices make the IoT face greater security threats [4,5,6,7]. These IoT devices are not only facing threats from outside the network (Internet), but also facing double threats from inside the network (between devices and devices, people and devices). Traditional network security protection methods are difficult to identify malicious behaviors of internal personnel. Therefore, new models and technologies are needed to detect insider threats in the IoT environment. The behavior-based insider threat detection system is an effective mechanism to defend the insider threat of the IoT. When an internal person has or will perform an attack, he/she may show some changes in the activities, which are the result of unmet expectations, dissatisfaction and social isolation [8,9,10].

There are many IoT devices around us, that can sense, store, compute, and communicate information, and insiders can easily commit data leakage, malware, and Denial-of-Service (DoS) attacks through IoT devices [11]. The identification methods for IoT devices belonging to insider threat actors can be built by anomaly detection for user’s behaviors of IoT devices based on application layer [12]. User behavior anomaly detection extracts user profiles and detects anomalies when current user behavior deviates from extracted user profiles. User profiles can be modeled by behavior data such as email, logon, web, file and print from the application layer of IoT devices.

What’s more, web traffic features are extracted and input into the classification algorithms, such as decision tree and random forest, to detect malicious web behaviors. However, the effectiveness relies on enough labeled samples which are difficult to collect due to the lack of malicious samples of the IoT insiders. In addition, web browsing behaviors have been studied for the e-business workflow and recommendation system, however these methods analyze the web browsing patterns within a specific server and cannot be applied directly in the IoT insider threat detection.

In this paper we firstly present the individual user behavior (IUAD) model that characterizes the dynamic change of user behavior based on power distribution to detect the deviation with respect to the usual normal behavioral change pattern of each user, as observed in a prolonged period of time, and then we propose the peer-group behavior (PGAD) model to evaluate the mutual behavioral similarity among users in one peer-group under the assumption that users in one peer group usually have common jobs and exhibit consistency of behavioral dynamic change. More specifically, to automatically identify the peer-group, the relationship network of users is built based on the co-occurrence of browsing behavior and then the peer groups are extracted from the relationship network of users using the dominant set framework. Furthermore, we develop the fusion model that combines IUAD model and PGAD model to detect insider threat from the perspectives of personal dynamic browsing anomaly and dynamic inconsistency among one peer group.

In summary, the proposed method makes the following main contributions.

- We propose an individual user behavior model (IUAD) to characterize the change of user browsing behavior based on power distribution under the assumption that the rate of individual user behavior change is continuous and user-specific.
- We put forward the peer-group behavior model (PGAD) model that automatically identifies the peer-group by applying the dominant set method to relationship network of users, and compare the mutual behavioral consistency among all users in the peer-group under the assumption that the user behavior is similar with his/her peers.
- We propose a fusion model for the insider threat detection that identifies the deviation of the individual dynamic behavior from two complementary perspectives: dynamic change of individual user behaviors and the dynamic inconsistency of user behaviors among all users in the peer-group.

## 2. Related Work

There are studies in different domains analyzing web browsing behaviors. Erdem et al. proposed an e-business workflow that is designed to generate test scripts based on user browsing behaviors. The e-business workflow is able to generate browsing patterns that are unseen in the historical web usage data [13]. Bhuvaneswari et al. proposed a density-based clustering algorithm that uses a non-Euclidean distance metric to compute the similarity between the users based on the sequence of web pages visited by them and the formed clusters are used to build the recommendation model [14]. Hawalah et al. modeled a user’s interests by mapping the content of web pages visited by the user to a reference ontology and the user’s interests can be used for personalized recommendation [15]. What’s more, the web queries that a user issues to search engines, are modeled based on temporal characteristics, including trends, periodicity, and surprise disruptions [16]. Based on the temporal characteristics of user behavior, the search engines can enhance query suggestions, crawling policies, and result ranking. These studies analyze web browsing behaviors for the e-business and recommendation system, and they cannot be applied directly in the IoT insider threat detection. Different from these studies, we analyze web browsing behaviors to detect IoT insider threat.

In addition, there are many studies detecting malicious web behaviors based on web traffic analysis. Al-Bataineh et al. proposed a classification algorithm to identify malicious data stealing attempts within web traffic. The classifier uses entropy and byte frequency distribution of HTTP POST request contents as features [17]. Ranjan et al. apply machine learning to predict malicious users from the legitimate users by using the traffic generated by users’ browsing on the web application. They used Random forest along with decision trees, binary classification, Clustering, and time series to compare the output to select the best-suited approach [18]. Vassio et al. build users’ profiles by tracking users from web traffic from a certain area, and show how to re-identify users in a future time based on their web behavior fingerprints [19]. These studies apply supervised learning algorithms and enough labeled dataset is necessary. However, the labeled dataset is difficult to collect due to the lack of malicious samples of IoT insiders. Unlike these studies, we adopt anomaly detection methods that don’t require malicious samples of IoT insiders.

A few works have been proposed to build user profile from user’s web browsing behavior for the purpose of user identification. From the perspective of page access frequency and page view time, four web user profile models [20], called TF-PVN, TF-PVT, TFIDF-PVN and TFIDF-PVT, are proposed to compute the weight of each domain name extracted from the history records of the target computer. Domains are ranked according to their weighting values and the top *N* domains from the target computer are chosen to form the profile vectors of the target computer. The candidate computer with the highest cosine similarity for the target computer has the highest probability that it is used by the same user. On the other hand, support-based and lift-based user profile models pick the top patterns from each user and union them to form the set of candidate patterns [21]. Then the within-user strength and the relative strength of candidate patterns are calculated to form the user profile vectors. User identification is conducted based on the Euclidean distance between two vectors. However, these methods model the behaviors by computing a fixed set of candidate patterns and ignore the continuously evolving of user behaviors. If the insider profile contains just static information, this eventually leads to disabling insider threat detection system due to generating many false positives as the user browsing behavior changes over time. By contrast, we take into consideration dynamic change of individual user behavior for insider threat detection.

## 3. The Proposed Model

**Figure 1:**
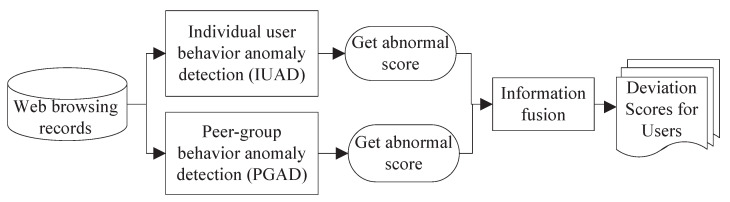
The proposed fusion framework for insider threat detection.

We propose an insider threat detection system that builds a fusion model from user web browsing behavior. The overall structure of the proposed model is illustrated in Figure 1. The model mainly contains three components that are named individual user behavior anomaly detection (IUAD), peer-group behavior anomaly detection (PGAD) and information fusion. We get abnormal scores from the first two components, and then fuse them to obtain the final abnormal scores in the third component.

To formalize the problem studied in this work, we will use the following notation. Let *U* be the set of users and *R* be the set of domain names accessed by users. And, let *Q* be the set of browsing records, such that q∈Q is a 3-tuple of the form q=<u,r,time>, where $u\in U={\left\{u_{j}\right\}}_{j\;=\;1}^{l}$, $r\in R={\left\{r_{g}\right\}}_{g\;=\;1}^{m}$, and time is the date the user accessed the domain name. For each user the contiguous browsing records can be divided into *n* different time periods based on fixed time span *c*, each time period consists of a few records, i.e., $e_{t}={\left\{q_{d}\right\}}_{d\;=\;v}^{v^{\prime }},q_{d}\in Q,\left|q_{v^{\prime }}.time-q_{v}.time\right|=c,c\in N^{+}$. Given *n* time periods for one user, the first *k* time periods ${\left\{e_{t}\right\}}_{t\;=\;1}^{k}$ are used for building the user profile and each one of the remaining time periods ${\left\{e_{t}\right\}}_{t\;=\;k+1}^{n}$ is scored based on the IUAD and PGAD, and then the two scores are combined as the ultimate result to detect whether there is an anomaly in the test time period. In this paper, we set the fixed time span as 1 day.

### 3.1. Individual User Behavior Anomaly Detection (IUAD)

**Figure 2:**
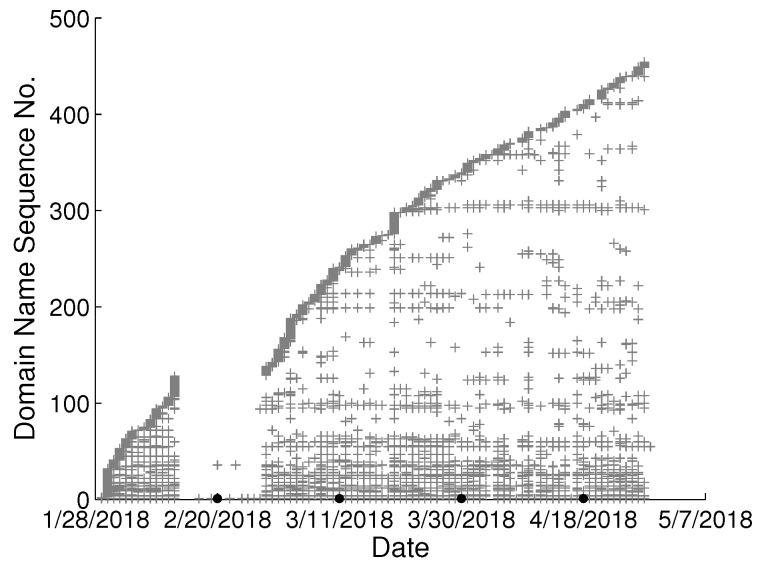
Domain names visited in chronological order from user A in our lab.

**Figure 3:**
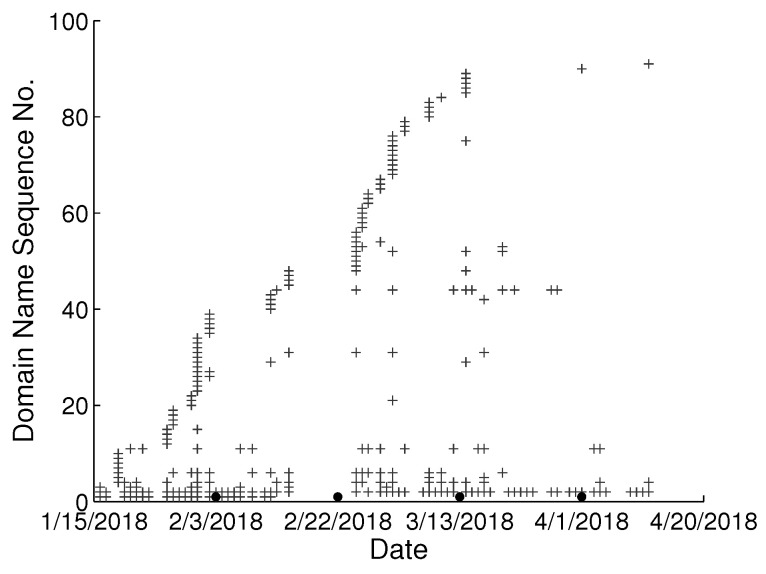
Domain names visited in chronological order from user B in our lab.

**Figure 4:**
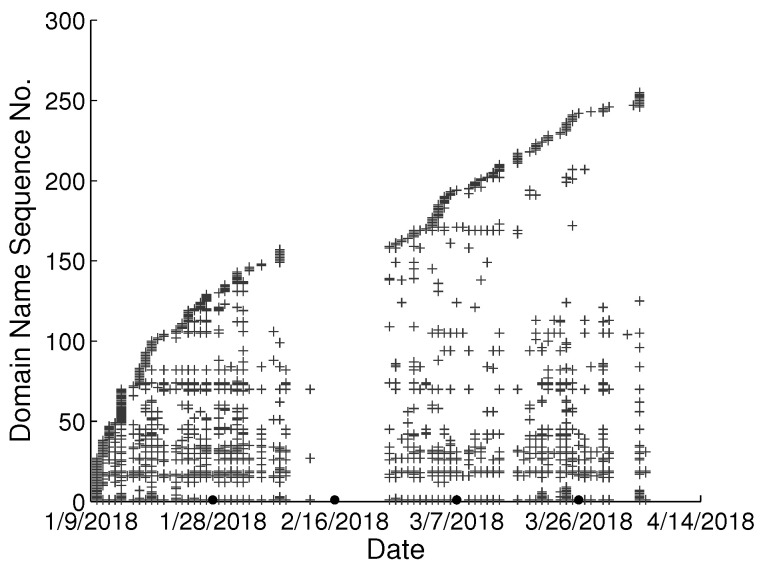
Domain names visited in chronological order from user C in our lab.

**Figure 5:**
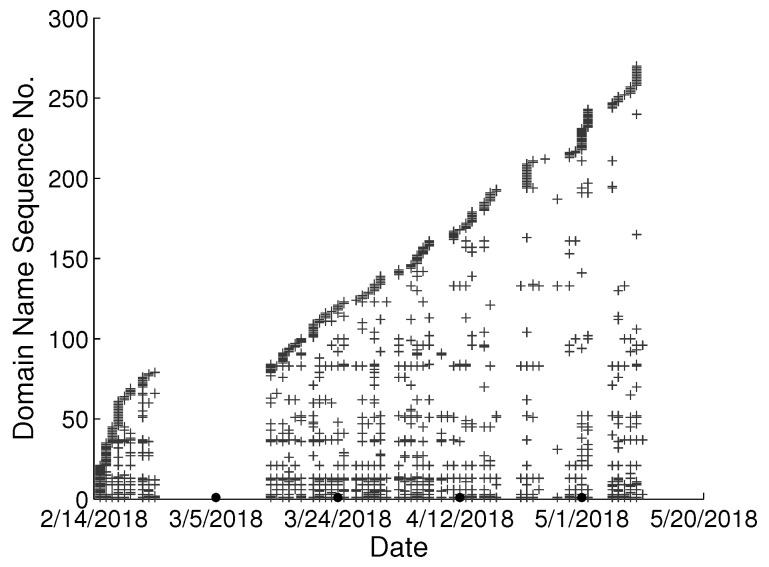
Domain names visited in chronological order from user D in our lab.

To study the behavioral change pattern of the individual user, we collect web browsing records from 4 users in our lab over the months of January to May 2018. The domain names visited in chronological order are presented in Figure 2, Figure 3, Figure 4 and Figure 5. We use various sequence numbers to denote different domain names. Horizontal axis represents the date and the vertical axis is the corresponding domain name sequence number visited on that day. The same sequence number in vertical axis of two sub-figures may represent the different domain names. A period without records occurs in every sub-figure due to winter vacation of our lab. From the figure, we can see new domain names are generated every day for each user and we assume the rate of generation is continuous and user-specific. Based on this assumption, we model the dynamics of visited domain names by each user as a power distribution. Consequently, the emerging domain name sequence number in the *t*th time period $e_{t}$ can be written as:

(1) $$f\left(t\right)=\alpha t^{\gamma }+b,1\leq t\leq k$$

where α,γ and *b* are parameters to learn by employing least-squares approximation.

Since the normal pattern of continuously evolving domain names is supposed to be the power distribution and is formalized in the Equation (1), the abnormal change of user behavior can be measured based on the deviation of emerging domain names from the power distribution. The abnormal score in the test time period can be written as:

(2) $$z_{t}=\left\{\begin{array}{cc} y\left(t\right)-f\left(t\right) & \;if\;y\left(t\right)>f\left(t\right)\\ 0 & otherwise \end{array}\right.,\;k<t\leq n$$

where $y\left(t\right)$ is the actual newest sequence number of domain name visited in the *t*th time period. We focus on the deviation when the actual sequence number is larger than the predicted one, and the larger the deviation the more anomaly the user behaviors. Because the insider’s malicious activities usually require accessing new resources that he has not visited before, we consider it normal if the actual sequence number is approximately equal to or less than the predicted one.

Finally, the abnormal scores of different test time periods for one user derived from the IUAD submodel can be represented as a vector: $Z_{IUAD}={\left(z_{t}\right)}_{1\times \left(n-k\right)},k<t\leq n$ and [k,n] is the set of indexes for different test time periods.

### 3.2. Peer-Group Behavior Anomaly Detection (PGAD)

Since users are not isolated in the insider system. One user’s behavior should be in line with his/her peers. In an insider threat detection context, the intuition is that user activity should reflect the user’s job role and users with similar job role should exhibit similar behavior in web browsing behavior. In this section, due to behavioral similarity among users in one peer-group, first we introduce the relationship network of users, second peer-groups are extracted from the network and we detect abnormal web user behaviors that are unusual compared to the user’s peers in the same peer-group. Instead of analyzing users independently in IUAD, PGAD analyzes user browsing behaviors that are not common to observe over the entire peer-group.

#### 3.2.1. Relationship Network of Users

**Figure 6:**
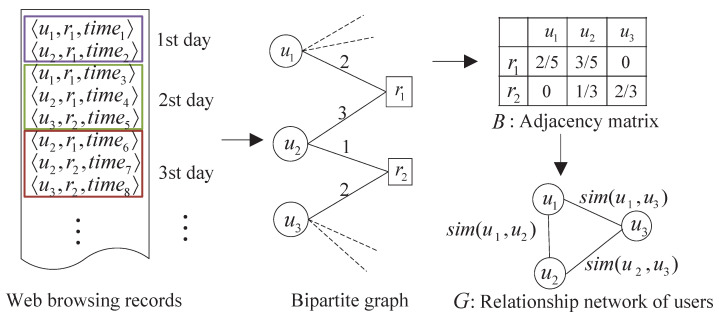
An example for the construction procedure of relationship network of users.

The relationship network of users is constructed as a completely connected undirected graph. The example procedure is illustrated in Figure 6. This undirected edge-weighted graph with no self-loops can be formulated as $G=\left(U,O,\lambda \right)$, where $U={\left\{u_{j}\right\}}_{j\;=\;1}^{l}$ is the set of users, O⊆U×U is the edge set that connect two users, and $\lambda :O\to R^{+}$ is the positive weight function. The weights on the edges of the graph are represented by a corresponding l×l symmetric similarity matrix $A=\left(a_{ij}\right)$ defined as:

(3) $$a_{ij}=\left\{\begin{array}{cc} sim\left(u_{i},u_{j}\right) & if\;i\neq j\\ 1 & otherwise \end{array}\right.$$

Here $sim\left(u_{i},u_{j}\right)$ reflects the similarity relationship between the user $u_{i}$ and $u_{j}$.

In order to obtain the distance between users, the users and visited domain names firstly are mapped onto a bipartite graph, such that users and domain names are modeled as vertices, and an edge represents the number of times that a user accessed the domain name. We summarize the information in this bipartite graph of users and domain names in an adjacency matrix *B* of size m×l, such that cell

(4) $$B_{gj}=\frac{count\left(\langle u_{j},r_{g},time\rangle \right)}{\sum_{\forall u_{p}\in U}count\left(\langle u_{p},r_{g},time\rangle \right)}$$

where $count\left(\langle u_{j},r_{g},time\rangle \right)$ is the number of access records that appear in a time period. The cells in this matrix are weighted according to inverse frequency; i.e., the importance of a domain name is inversely proportionally to the number of users that access this record (e.g., domain names visited by 2 users contribute 0.5, and by 3 users contribute 0.33). The adjacency matrix summarizes the frequency with which a user accesses a domain name in a time period.

Based on the adjacency matrix computed from the records in the first *k* periods, the distance between two users can be represented by a corresponding l×l symmetric matrix $D=\left(d_{ij}\right)$ defined as:

(5) $$d_{ij}=\left\{\begin{array}{cc} \sqrt{\sum_{1⩽g⩽m}{\left(B_{gi}-B_{gj}\right)}^{2}} & if\;i\neq j\\ 0 & otherwise \end{array}\right.$$

And then the distance is transformed into similarity (edge-weight) using a Gaussian kernel and the symmetric similarity matrix $A=\left(a_{ij}\right)$ in the Equation (3) is rewritten as:

(6) $$a_{ij}=\left\{\begin{array}{cc} e^{-\frac{d_{ij}}{\sigma }} & if\;i\neq j\\ 1 & otherwise \end{array}\right.$$

where σ>0 is the variances of the distance between two users.

#### 3.2.2. Anomaly Based on Behavioral Similarity

To automatically discover the peer-group, we adopt the dominant set technique [22] to iteratively find maximally a group of similar users in the edge-weighted graph. For a non-empty subset of users $S\subseteq U,u_{i}\in S$ and $u_{j}\notin S$, the relative similarity between user $u_{i}$ and $u_{j}$ is defined as:

(7) $$\Phi_{S}\left(i,j\right)=a_{ij}-\frac{1}{\left|S\right|}\sum_{u_{p}\in S}a_{ip}$$

Next, the overall similarity between user $u_{i}$ and the users of $S\backslash \{u_{i}\}$ is defined as follows:

(8) $$\varphi_{S}\left(i\right)=\left\{\begin{array}{cc} 1 & if\;\left|S\right|=1\\ \sum_{u_{j}\in S\backslash \left\{u_{i}\right\}}\Phi_{S\backslash \{u_{i}\}}\left(j,i\right)\varphi_{S\backslash \{u_{i}\}}\left(j\right) & otherwise \end{array}\right.$$

A non-empty subset of users S⊆U is said to be a dominant set (peer-group) if:

1. $\varphi_{S}\left(i\right)>0$, for all $u_{i}\in S$
2. $\varphi_{S⋃\{u_{i}\}}\left(i\right)<0$, for all $u_{i}\notin S$

On account of the behavioral similarity among users in the peer-group, the abnormal user behavior can be measured based on the user’s behavioral deviation from his peers’. Correspondingly, we score the user $u_{i}$ with his peers $S\backslash \{u_{i}\}$ in the test time period as:

(9) $$z_{t}^{\prime }={\left|S\right|}^{-1}\sum_{u_{p}\in S}\left(d_{ip}^{t}-k^{-1}\sum_{t^{\prime }=1}^{k}d_{ip}^{t^{\prime }}\right),\;k<t\leq n$$

where $d_{ip}^{t}$ and $d_{ip}^{t^{\prime }}$ can be obtained based on the Equation (5) with the browsing records in the test time period and in the history respectively. The larger the score, the more anomaly the user behavior.

Finally, the abnormal scores of different test time periods for one user derived from the PGAD sub-model can be represented as a vector: $Z_{PGAD}={\left(z_{t}^{\prime }\right)}_{1\times \left(n-k\right)},\;k<t\leq n$ and [k,n] is the set of indexes for different test time periods.

### 3.3. Information Fusion

In this section, we propose the fusion method based on IUAD and PGAD to comprehensively consider the behavioral change of individual user and behavioral consistency among users in the peer-group for abnormal behavioral detection in enterprise networks.

One of the issues for fusion is that the scores are generated from different mechanisms and as a result, provide no common ground for comparison. To counter this, the scores firstly have to be normalized.

(10) $$\begin{array}{cc} a_{t} & =\frac{z_{t}-min\left(Z_{IUAD}\right)}{max\left(Z_{IUAD}\right)-min\left(Z_{IUAD}\right)}\\ a_{t}^{\prime } & =\frac{z_{t}^{\prime }-min\left(Z_{PGAD}\right)}{max\left(Z_{PGAD}\right)-min\left(Z_{PGAD}\right)} \end{array}$$

where k<t⩽n.

Subsequently, we formulate the fusion scheme as a weighted linear combination of the IUAD and PGAD:

(11) $$h_{t}=\left(1-w\right)\cdot a_{t}+w\cdot a_{t}^{\prime }\;,\;k<t\leq n$$

where w∈[0,1] is the trade-off factor for IUAD and PGAD sub-models. When w=1, only PGAD is used; and when *w* = 0, only IUAD is used. Consequently, the finally abnormal scores of different test time periods for one user from the fusion method can be represented as a vector: $H={\left(h_{t}\right)}_{1\times \left(n-k\right)},\;k<t\leq n$. If one score is larger than a specific threshold, the corresponding test time period is labeled as an anomaly.

## 4. Experiments

### 4.1. Dataset

Due to the lack of availability of proper web browsing logs in enterprise network, we utilize an insider threat dataset published by CERT Carnegie Mellon University for this research. The 17-month period dataset “R4.2.tar.bz” consists of five types of records of 1000 employees, i.e., HTTP, File, device, logon, email records. All HTTP records are chosen for this analysis. Each record contains user, PC, URL and web page content with time stamps. The web page content is ignored and an HTTP record is depicted in Table 1. Meanwhile, user browsing anomalies are simulated based on an attack scenario where insiders begin surfing job websites and soliciting employment from a competitor, and then they use a thumb drive to steal data before leaving the company.

### 4.2. Experiment Design

#### 4.2.1. Construction and Validation Sets

First of all, we divide all HTTP records into a number of samples and every sample is composed of records of one day. And then we split these samples into two different sets: construction and validation. The construction set is composed of a certain percentage of the samples and is used to create and train user behavior model. The validation set consists of the remaining percentage of the samples and the full set of user browsing behavior anomalies, and is used to yield a detection performance. Specifically, for each experiment, we split the dataset with different percentages for both sets, construction and validation, namely: 80–20, 70–30, 50–50, 30–70, and 20–80, to make a sufficient comparison.

#### 4.2.2. Threshold

To emit an evaluation, we compare the abnormal scores of the samples in the validation set against a threshold. A sample is labeled as anomaly, if its score is higher than the threshold, and normal, otherwise. We vary the threshold from 0 to 1 to study the performance of our model, thereby drawing a so-called Receiver Operating Characteristic (ROC) curve. So, we start with a low threshold, getting a lax model; then, we increase the threshold slowly until we get a very strict one. Doing so, we have got results from 100% False Positive Rate (FPR) with 0% False Negative Rate (FNR), to 0% FPR with 100% FNR.

### 4.3. Evaluation Metrics

In order to compare detection models one another, we have used two different measurements: Area-Under-the-Curve (AUC) and the Minimum Misclassification Point (MMP). AUC denotes the area under a ROC curve. An AUC equal to one amounts to the perfect model, which correctly marks every sample, as normal or abnormal. Conversely, an AUC equal to zero corresponds to the worst model ever. What’s more, MMP denotes a point in the ROC curve that minimizes FPR+FNR. The smaller the MMP, the better the detection model.

In order to process the large experimental dataset and get the experimental results quickly and effectively, we choose a Linux server Dell R740 as the experimental platform and the experimental simulation environment in this paper was follows: Ubuntu 20.04 64-bit, 125 G RAM, Python 3.7. The configuration of the server can provide an ideal environment for the running of the experimental Python programs.

### 4.4. Overall Anomaly Detection Results

In this section we evaluate the performance of our fusion model in detecting browsing behavior anomaly. We vary the hyper-parameters *w*, which is the trade-off term for combining IUAD and PGAD sub-models. The result is shown in Table 2 and a number in bold means that the corresponding method performs better than its counterpart does, for the same metric and same experiment dataset.

Looking closely at the table, we conclude that the fusion model outperforms the two single sub-models IUAD and PGAD, since the central part of Table 2, which corresponds to the fusion method, contains a majority amount of bold typeface numbers. What’s more, the best performance is obtained when we vary *w* between 0.1 and 0.3, at which both sub-models are combined most appropriately. Besides, we can notice that as we shorten the amount of available training, the fusion method is most stable compared to the two sub-models from the perspective of standard deviation of the MMP and achieves the best AUC value for the 70–30 (%) experiment.

**Figure 7:**
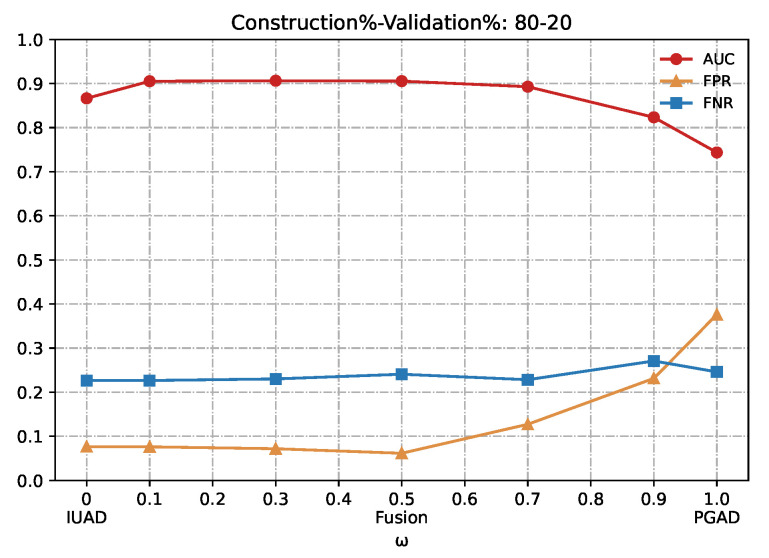
Detection results of the IUAD, the PGAD and the fusion model in terms of AUC, FPR, FNR (User’s http records division based on Construction%–Validation%: 80%–20%).

**Figure 8:**
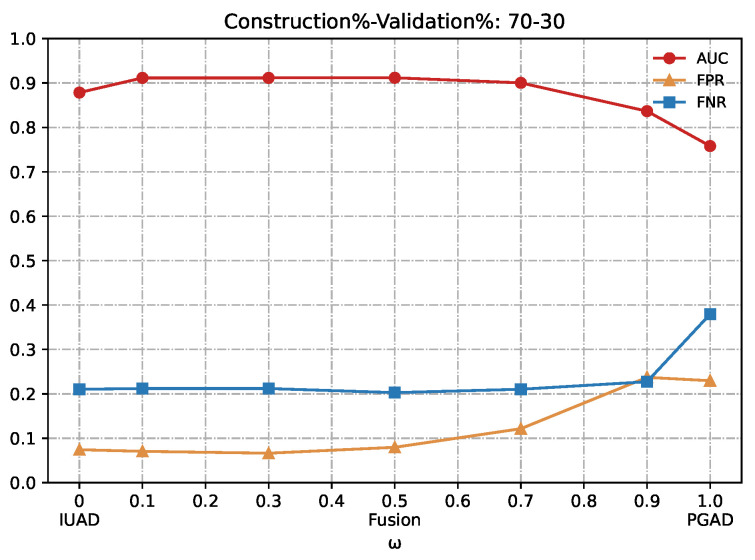
Detection results of the IUAD, the PGAD and the fusion model in terms of AUC, FPR, FNR (User’s http records division based on Construction%–Validation%: 70%–30%).

**Figure 9:**
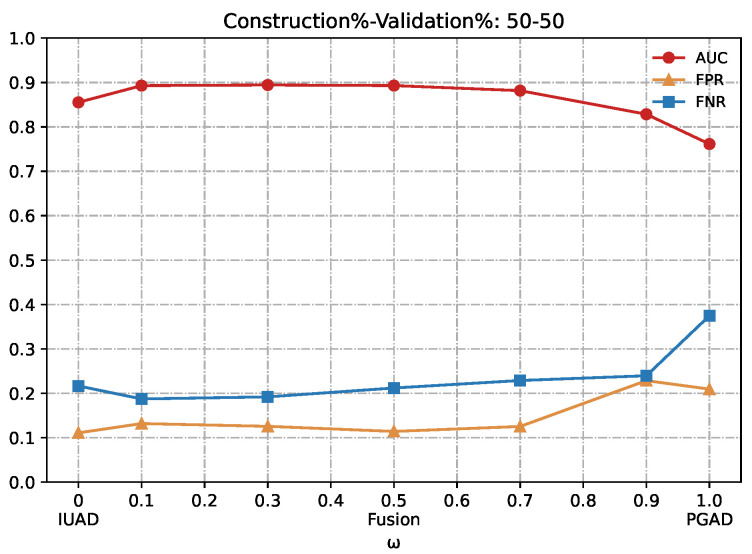
Detection results of the IUAD, the PGAD and the fusion model in terms of AUC, FPR, FNR (User’s http records division based on Construction%–Validation%: 50%–50%).

**Figure 10:**
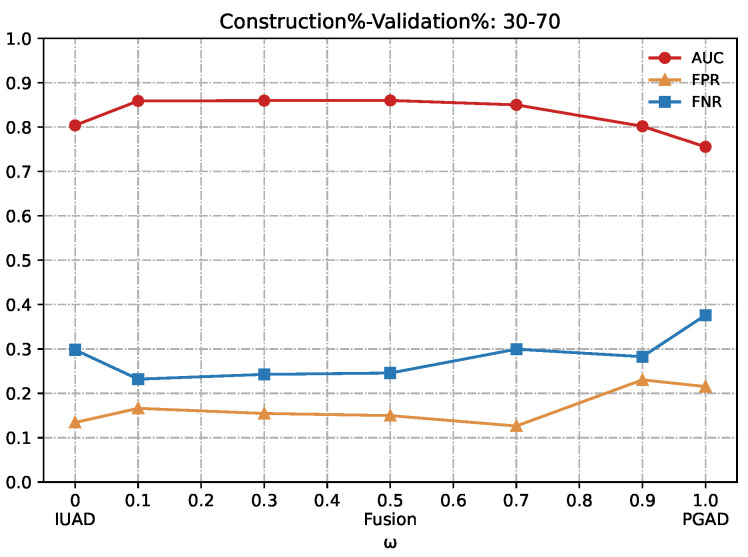
Detection results of the IUAD, the PGAD and the fusion model in terms of AUC, FPR, FNR (User’s http records division based on Construction%–Validation%: 30%–70%).

**Figure 11:**
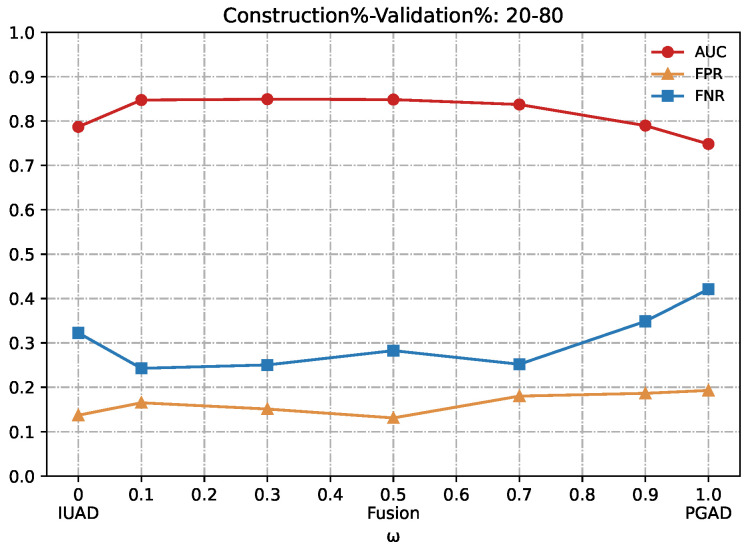
Detection results of the IUAD, the PGAD and the Fusion model in terms of AUC, FPR, FNR (User’s http records division based on Construction%–Validation%: 20%–80%).

We can clearly appreciate in Figure 7, Figure 8, Figure 9, Figure 10 and Figure 11 that the combination based on IUAD and PGAD supersedes single IUAD and single PGAD. AUC, FPR and FNR are the metrics compared in each of the graphs. We can see the metrics achieve best when the combination rate *w* is set to 0.1 and 0.3 whenever the amount of available training in different graphs.

Summarizing, the fusion method achieves better performance than IUAD and PGAD that validates the effectiveness of our fusion scheme and the complementary capacity of two sub-models. Based on the combining factor between IUAD and PGAD, the fusion method can capture user browsing behavior from both the dynamic change of individual user and behavioral similarity among users in the same peer-group.

### 4.5. Comparison Study

For comparison purposes, we have implemented and tested TF-PVN [20], TFIDF-PVN [20], support-based user profile [21] and lift-based user profile [21]. These four models build user browsing behaviors for user identification, but they can be extended to detect abnormal behaviors with a little modification.

TF-PVN and TFIDF-PVN choose top *N* domains from the target computer to form the profile vectors of the target computer and then apply the cosine similarity measure to assess the similarity of two browsing histories. In our experiment, we select the top one-sixth of the domains as suggested in the TF-PVN and TFIDF-PVN and employ the cosine similarity measure to assess the anomaly of the user current behaviors compared to the past activity. While, support-based and lift-based user profile pick the top patterns from each user and union them to form the set of candidate patterns. The distance between two profiles is computed based on Euclidean distance. In our experiment, we choose top 10 domains from each user as suggested in the support-based and lift-based user profile method and use the Euclidean distance to assess the abnormal score of the user’s current behaviors based on the history browsing records.

**Figure 12:**
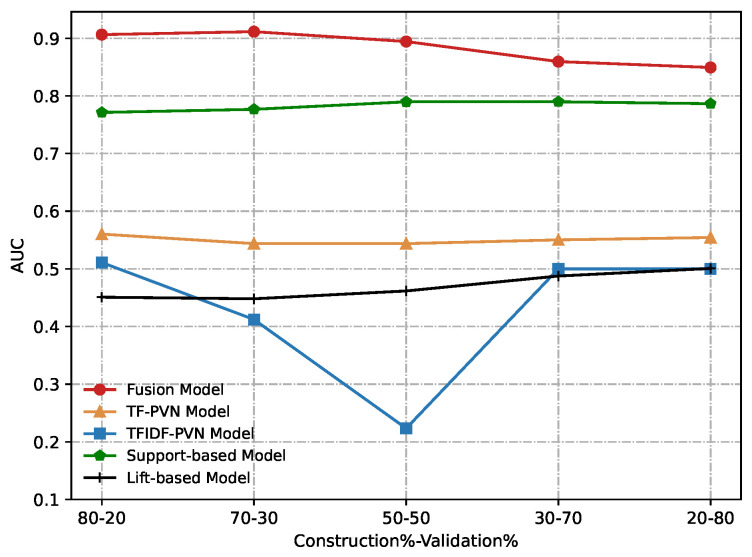
Comparative results of the Fusion model, TF-PVN model, TFIDF-PVN model, Support-based model and Lift-based model in terms of AUC.

**Figure 13:**
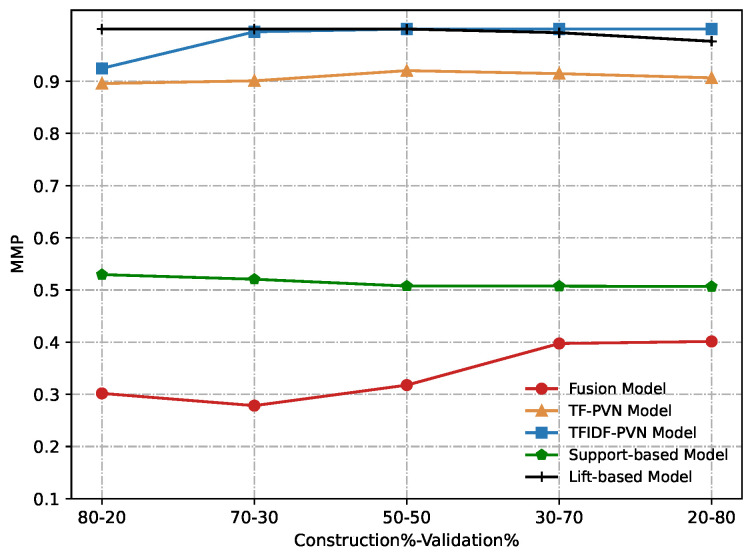
Comparative results of the Fusion model, TF-PVN model, TFIDF-PVN model, Support-based model and Lift-based model in terms of MMP.

Due to the best performance, we use w=0.3 in the fusion method for comparison. Figure 12 and Figure 13 show our experimental results obtained from conducting a comparative evaluation of all these methods on different construction and validation datasets according to AUC and MMP. From these figures, we can notice that our proposed the fusion model significantly outperforms other methods compared in any metric.

Table 3 summarizes our experimental comparison. As before, we use bold typeface to stress best results, and follow the same convention in the confection of the table. From the table, we conclude that our fusion method outperforms the others. For example, the top of Table 3, which corresponds to our fusion method, contains a majority amount of bold typeface numbers. Further, Table 3 shows that in six out of seven performance indicators, our fusion method surpasses the others with significant statistical differences. This can be explained by that the other methods are all based on a fixed set of candidate patterns and ignore the continuously evolving of user browsing behaviors, and the behavioral similarity among users in the peer-group is not considered. Hence they fail to uncover the abnormal user behaviors because of high false positives.

## 5. Conclusions

With the growth of IoT, new security challenges arise in the existing security frameworks. Because internal staff can easily access the network environment through IoT devices, it is a great challenge to detect internal attacks. In the paper, an insider threat detection model is introduced, which models user web browsing behavior. At the same time, the model is a comprehensive model, which detects the deviation from the power distribution of individual user browsing behavior and behavioral similarity among users in the peer-group to the user’s current behavior simultaneously. To automatically identify peer-group in an organization, relationship network of users is constructed based on an adjacency matrix that summarizes the frequency with which a user accesses a domain name in the history, and then the dominant set technique is applied into the relationship network of users to uncover a group of users with similar browsing behavior as one peer-group. After a wide range of experiments, it is verified that the proposed fusion model obtains best performance when we set the trade-off actor for combining IUAD and PGAD sub-models as w=0.1 and w=0.3, at which both sub-models are combined most appropriately. Compared with other models, the fusion method proposed in the paper could accurately detect the abnormal behavior of internal users. There are some insider threats on IoT devices that are not connected to the monitored network, such as taking photos of sensitive information, remain outside the detection range. Therefore, the future researchers, including us, will devise breakthrough ideas to extend and enhance current security systems for IoT insider threat prevention.

## Figures and Tables

**Table 1:** A HTTP record in the dataset.

ID	V1Y4-S2IR20QU-6154HFXJ
Date	01/02/2010 06:55:16
User	LRR0148
PC	PC-4275
URL	http://msn.com/The_Human_Centipede_First_Sequence/katsuro/arjf309875127.htm

**Table 2:** Overall anomaly detection performance in terms of AUC and MMP. A number in bold means the corresponding method performs best.

Construction%–Validation%			80–20	70–30	50–50	30–70	20–80	Avg.	Std.
IUAD Sub-model (w=0)	AUC		0.8664	0.8784	0.8553	0.8038	0.7870	0.8382	0.0403
	MMP	FPR	**0.0764**	0.0743	0.1110	0.1344	0.1370	0.1066	0.0303
		FNR	**0.2265**	0.2104	0.2166	0.2980	0.3226	0.2548	0.0517
PGAD Sub-model (w=1)	AUC		0.7436	0.7580	0.7614	0.7554	0.7484	0.7534	**0.0072**
	MMP	FPR	0.3761	0.2296	0.2095	0.2153	0.1929	0.2447	0.0746
		FNR	0.2460	0.3794	0.3748	0.3763	0.4209	0.3595	0.0663
Fusion Model (w=0.1)	AUC		**0.9052**	**0.9114**	**0.8929**	**0.8588**	**0.8475**	**0.8832**	0.0285
	MMP	FPR	**0.0761**	0.0706	**0.1319**	**0.1662**	**0.1651**	0.1220	0.0465
		FNR	**0.2265**	0.2120	**0.1874**	**0.2320**	**0.2427**	0.2201	0.0214
Fusion Model (w=0.3)	AUC		**0.9063**	**0.9114**	**0.8943**	**0.8595**	**0.8493**	**0.8842**	0.0281
	MMP	FPR	**0.0718**	**0.0664**	**0.1257**	**0.1547**	**0.1510**	**0.1139**	0.0425
		FNR	**0.2301**	**0.2120**	**0.1920**	**0.2427**	**0.2504**	**0.2254**	0.0237
Fusion Model (w=0.5)	AUC		**0.9054**	**0.9117**	**0.8930**	**0.8599**	**0.8485**	**0.8837**	0.0280
	MMP	FPR	**0.0615**	0.0797	0.1142	0.1499	0.1309	0.1072	0.0363
		FNR	**0.2407**	0.2028	0.2120	0.2458	0.2826	0.2368	0.0315
Fusion Model (w=0.7)	AUC		0.8928	0.9002	0.8816	0.8499	0.8374	0.8724	0.0274
	MMP	FPR	0.1272	0.1216	0.1254	0.1264	0.1801	0.1361	**0.0247**
		FNR	0.2283	0.2104	0.2289	0.2995	0.2519	0.2438	**0.0344**
Fusion Model (w=0.9)	AUC		0.8234	0.8365	0.8285	0.8017	0.7898	0.8160	0.0195
	MMP	FPR	0.2313	0.2372	0.2286	0.2304	0.1864	0.2228	0.0206
		FNR	0.2708	0.2273	0.2396	0.2826	0.3487	0.2738	0.0475

**Table 3:** Comparison study in terms of AUC and MMP. A number in bold means the corresponding method performs best.

Construction%–Validation%			80–20	70–30	50–50	30–70	20–80	Avg.	Std.
Fusion Model (w=0.3)	AUC		**0.9063**	**0.9114**	**0.8943**	**0.8595**	**0.8493**	**0.8842**	0.0281
	MMP	FPR	**0.0718**	**0.0664**	**0.1257**	**0.1547**	**0.1510**	**0.1139**	0.0425
		FNR	**0.2301**	**0.2120**	**0.1920**	**0.2427**	**0.2504**	**0.2254**	0.0237
TF-PVN Model	AUC		0.5603	0.5438	0.5438	0.5503	0.5544	0.5505	**0.0071**
	MMP	FPR	0.6089	0.6141	0.6561	0.6964	0.5409	0.6233	0.0581
		FNR	0.2867	0.3041	0.2642	0.2181	0.3656	0.2877	0.0542
TFIDF-PVN Model	AUC		0.5110	0.4122	0.2234	0.5000	0.5000	0.4293	0.1218
	MMP	FPR	0.3920	0.0608	0	0	0	0.0906	0.1706
		FNR	0.5327	0.9339	1	1	1	0.8933	0.2036
Support-based Model	AUC		0.7713	0.7767	0.7897	0.7898	0.7865	0.7828	0.0084
	MMP	FPR	0.3720	0.3731	0.3324	0.3569	0.3561	0.3581	**0.0165**
		FNR	0.1575	0.1475	0.1751	0.1505	0.1505	0.1562	**0.0112**
Lift-based Model	AUC		0.4509	0.4481	0.4617	0.4876	0.5008	0.4698	0.0233
	MMP	FPR	1	1	1	0.3571	0.3636	0.7441	0.3504
		FNR	0	0	0	0.6359	0.6129	0.2498	0.3421

## Data Availability

The data in this study are available.
